# Supplementary material for: Using smartphones to study vaccination decisions in the wild
Source: PLOS Digit Health. 2024 Aug 8;3(8):e0000550. doi: 10.1371/journal.pdig.0000550 (PMC11309433; doi:10.1371/journal.pdig.0000550)
Supplement: S1 Appendix — Document containing statistics of infected, vaccinated and seeds of each round. (PDF) [file pdig.0000550.s001.pdf]

Supporting Information for

Using smartphones to study vaccination decisions in the wild

Nicolò Alessandro Girardini, Arkadiusz Stopczynski, Olga Baranov, Cornelia Betsch, Dirk Brockmann, Sune Lehmann, Robert Böhm

S1 Rounds statistics

In each of the game rounds, the players were selected to be infected at the start of the round with a probability of $p = 0.015$. These could then spread the disease to the other players. As seen in table Table S1, there were issues with instantiating the seeds in waves *5, 6* and *8*, which then resulted in less infected participants during these rounds. Fortunately, the impact on the vaccination behaviors is limited.

During the first round there was quite a high vaccination rate, as players did not have direct experience with the game. We see that there is a general decline in the vaccine uptake, but we do not see dips after rounds that reported the issue with the initial seeds. We think that this, along with our choice to group all the observation relative to a single feedback condition, does not hinder the analysis of the vaccination behavior of the participants.

Table S1: **Seeds, infected and vaccinated per round**

Round	Seeds	Infected Rate	Vaccinated Rate
1	11	32.0%	43.4%
2	6	21.5%	43.5%
3	15	25.8%	35.9%
4	12	21.4%	35.9%
5	3	1.0%	40.5%
6	3	5.2%	33.2%
7	9	27.4%	29.5%
8	2	1.0%	29.9%
9	6	4.0%	30.6%
10	8	19.1%	27.0%
11	9	12.4%	29.2%
12	6	13.9%	26.2%
